# An Approach to Establishing International Quality Standards for Medical Travel

**DOI:** 10.3389/fpubh.2016.00029

**Published:** 2016-03-04

**Authors:** Ondřej Kácha, Beáta E. Kovács, Cormac McCarthy, Angela A. T. Schuurmans, Christopher Dobyns, Elisa Haller, Saba Hinrichs, Kai Ruggeri

**Affiliations:** ^1^Department of Psychology, Faculty of Arts, Masaryk University, Brno, Czech Republic; ^2^Institute of Neuroscience and Psychology, University of Glasgow, Glasgow, UK; ^3^Mary Immaculate College, Limerick, Ireland; ^4^Behavioural Science Institute, Radboud University Nijmegen, Nijmegen, Netherlands; ^5^Department of Politics and International Relations, University of Oxford, Oxford, UK; ^6^Engineering Design Centre, University of Cambridge, Cambridge, UK; ^7^The Policy Institute, King’s College London, London, UK; ^8^Policy Research Group, Department of Psychology, University of Cambridge, Cambridge, UK

**Keywords:** health care, medical travel, international quality standards, public policy, medical insurance, patient centeredness

## Abstract

The number of individuals traveling abroad is increasing annually. The rising popularity of medical travel and the absence of clear minimum quality requirements in this area urgently call for the development of international standards to ensure good practice and patient safety. The aim of this study is to identify the key domains in medical travel where quality standards should be established. Drawing from the evidence-based OECD framework and an extensive literature review, this study proposes three critical areas for consideration: minimum standards of health-care facilities and third-party agencies, financial responsibility, and patient centeredness. Several cultural challenges have been introduced that may pose a barrier to development of the guidelines and should be taken into consideration. Establishing international quality standards in medical travel enhances benefits to patients and providers, which is an urgent necessity given the rapid growth in this industry.

## Introduction

Medical travel is defined as the crossing of national borders with the purpose of receiving treatment that has been determined as essential to maintaining quality of life by a health professional, but may not need to be performed urgently (1). Medical travel should be distinguished from medical tourism, where the latter’s focus is on non-urgent and unnecessary treatments (e.g., cosmetic surgeries) or traveling for recreational and leisure purposes such as a spa visit (2). A strong international market for medical travelers has been established in recent years, consisting of informal patient networks and formal networks of medical providers (3). However, there are limited international guidelines currently in place to promote safe and effective medical travel, and issues arise regarding the quality of care as well as how standards may be achieved and maintained from one country to the next. Considering the general absence of medical travel regulations globally, the establishment of international quality standards is imperative to ensure the safety of patients and providers.

### Quality Standards

Although some individual countries offer definitions for standards of quality health care, there appears to be no universal definition for international quality standards (4). Broadly speaking, quality standards for health care can be considered the metric against which the performance of a service can be measured, which may be either “essential” – the absolute minimum to ensure safe and effective practice – or “developmental” – designed to encourage and support a move to better practice (5, 6). Thus, establishing a framework for essential international quality standards would provide a benchmark against which to measure all health-care services involved in medical travel.

Despite the absence of a universal definition, several guidelines exist regarding quality in the health-care context on clinical, national, and international levels. The Institute of Medicine [IoM; (7)] and the World Health Organization [WHO; (8)] tasked with the improvement of health-care services established working definitions and conceptual frameworks that encapsulate quality in health care. Additionally, the Organization for Economic Cooperation and Development [OECD; (9)] offers a comprehensive framework for the promotion of quality dimensions, which is based on findings by the Health Care Quality Indicators Project, and enables measurement, comparison, and achievement of uniformity on a national level.

There are, however, a number of limitations regarding the applicability of these existing guidelines within a medical travel context, particularly when it comes to global medical standards. Currently, the international guidelines are not reinforced by legislation; and therefore, health-care providers are not required to facilitate these standards in practice. The guidelines also do not provide comprehensive instructions on how to implement, monitor, or supervise the standards. Additionally, the OECD guidelines do not consider individuals who travel abroad for medically necessary care. Incorporating medical travel into the OECD guidelines will ensure a more comprehensive perspective of quality of medical care, taking into account the key motivations of medical travelers.

### Why Do Individuals Travel for Care?

Quality of care, cost, and access are among the key driving factors that influence a patient’s decision to travel for obtaining medical care (10–12). In an extensive review that examined studies of UK patients’ health-care preferences, a substantial number of questionnaires and surveys indicated the significant impact that quality, affordability, and access have on medical travelers’ choice of health-care facilities (11). Horowitz et al. (13) found that individuals traveling for essential procedures from industrialized countries were attracted to less industrialized countries by the low cost of care. Additionally, an increasing number of medical travelers from North America and Europe are accessing treatment in countries such as India and Brazil due to long waiting lists and bureaucracy (14). Medical travelers also appear to be frustrated with the restricted availability of facilities in their native countries and turn to more advanced health-care systems abroad which have been recognized as having state-of-the-art technology and high overall medical care [e.g., Thailand; (15)]. As such, it is recommended that those proposing and implementing international quality standards for medical travel consider the roles of quality of care, cost, and access.

## Proposed Approach

This paper offers a potential approach to establishing international quality standards for the rapidly expanding industry of medical travel. This approach addresses the challenges and issues, identified by conducting a scoping of the available literature considered most important in the design and implementation of frameworks for these standards. A two-step process is proposed to establish international quality standards in medical travel.

Given that the OECD framework already incorporates the domains of quality, cost, and access, there is a stable base for the development of international quality standards for medical travel. Subsequently, the OECD framework should be adapted to incorporate specific medical travel considerations (minimum standards of care and resources, insurance and cost considerations, and maintenance of a patient-centered approach). It has been suggested that the development standards should be naturally linked to the WHO as the pre-eminent global health policy institution (16). However – given that medical travel is largely considered as an economic intervention to control costs of health care – the OECD appears to be the most competent body to promote standard development, measurement, and further applications.

Second, the application of the standards could eventually benefit from independent review *via* a non-governmental body [such as the International Organization for Standardization (ISO)]. This offers considerable oversight toward maintenance of guidelines and standards. The selected organization should ensure that medical travelers receive care of appropriate quality, independent of where they are traveling. As this article focuses on establishing the standards in the first place, it is beyond its scope to detail all relevant considerations, though the establishment of the standards alone would provide medical travelers with an explicit statement of good practice, which covers a variety of potential risks and creates an opportunity for such access to expand responsibly.

## Main Domains

The main domains that need to be incorporated within an international quality framework for medical travel include minimum standards of care and resources, insurance and cost considerations, and maintaining a patient-centered approach across cultural boundaries. These domains were identified as the most salient based on scoping the existing literature that discusses quality challenges in the context of medical travel.

### Minimum Standards

As quality is one of the key factors on which patients base their treatment decisions, it is vital to ensure that health-care facilities operating on an international medical market meet a minimum quality standard to inform potential patients about their medical care options.

In contextualizing any minimum standard, a geographical location should be considered. As the OECD proposes that quality standards should be partly subject to countries’ differences (9), the country level is not sufficient. Rather, the standards should be adjusted to regions of particular countries and, where applicable, stratified by additional factors such as economic development, political stability, and local clinical standards.

Independent, external peer review is necessary to ensure that a particular health-care facility meets the minimum quality standards (17, 18). Within the medical travel market, a growing number of private organizations offer peer-review services to hospitals and other health-care facilities. For example, the International Medical Travel Journal lists more than 12 bodies offering this service (19). However, large numbers of providers may result in striking differences between the peer-reviewed facilities around the world.

To resolve this discrepancy, organizations providing international accreditation should be liable to a higher authority that would oversee the peer-review process of health-care facilities. The International Society for Quality in Health Care (ISQua) possesses the resources to take up this role (20). However, it is important to emphasize that even with a consistent accreditation system, accreditation does not equate to high quality but rather refers to the minimum quality standards (21, 22).

Furthermore, the cost of peer-reviewing services must be considered. While accreditation may be relatively affordable in developed countries, facilities grounded in low-level income areas may find it more difficult to engage with the accreditation process due to limited financial resources (23).

Apart from health-care facilities, third-party agencies that mediate the process of receiving care abroad have a substantial impact on quality standards within the medical travel industry (18). Given their role, they should also be subject to an evaluative process to ensure minimum standards of good practice, e.g., conformity to the legislation of sending and receiving country and confidential handling of patients’ data (18). Quality standards may also be supported by restricting the agencies to offer only health-care facilities that have been proven to meet the minimum standards. While entities such as the Joint Commission International (JCI) and Health On the Net Foundation (HON) have already made the initial steps in addressing this issue, this presents only a starting point toward the wider needs.

### Financial Responsibility

Serious consideration should be given to insurance and costs in medical travel (13), including all pre-, intra-, and post-operative care in both the destination country and country of origin. Medical travel companies and medical travel insurance do not usually cover treatment costs that occur before or after a given medical procedure, posing a burden on origin countries’ health resources that need to cover any necessary care beyond the initial operation. To reduce the improper load that is put on the countries of origin, medical travel insurance should account both for medical travel and pre- and post-treatment complications (18). Additionally, making insurance companies partially responsible for the compensation of patients in cases of accidents, postsurgery complications, or neglect could provide them incentive to cooperate exclusively with health-care facilities meeting the minimum standards (18).

### Patient Centeredness

Most definitions of quality in health-care services refer to patient centeredness, a concept of care where the overall well-being of the patient is prioritized (24). This includes adequate communication with the patient, information provision, transparency in quality of services, and opportunities for feedback. It is crucial for the destination hospital to inform potential patients about their facilities, exact procedure and possible outcomes, as well as communication about patient’s medical history (25).

Transparency refers to making accurate and useful information about performance and outcomes available to staff, patients, the public, and regulators (26). A transparent system will allow those involved, to publicly share their experiences (e.g., through freely accessible online database). Medical travel companies as well as destination clinics and hospitals would then have an (additional incentive) interest in increasing their quality of care for future patients. Finally, to ensure that these standards have been met regarding patient focus, any qualified clinical institution should be required to follow-up on patient experiences with health services in order to promote continued progression from minimum quality standards. This could include, but is not limited to, evaluation forms after operation and during hospital stay, as well as opportunity for complaints. Patient centeredness should become a global characteristic of all institutions that maintain the minimum standards of quality of care, thus helping to minimize complications and increase travelers’ well-being.

## Possible Cultural Challenges

Further challenges in the development of the standards revolve around linguistic and religious differences. Communication is likely to be impeded when doctor and patient do not speak the same native language, which may contribute to adverse events and medical errors (27–29). A study among Hispanic patients in the US has reported that patients list language as the most important variable for treatment satisfaction, even more important than ethnicity itself (30). To bridge the communication gap between practitioners and foreign patients, professional interpreters might be included in the medical process (31, 32).

Another significant barrier to establishing international quality standards lies in the area of religious and spiritual differences. These differences – capable of changing the course of treatment completely (33) – may not only occur among patients but also with physicians and those providing medical care (34). The distinct differences in perceptions of medical treatment from one religious group to another should be respected and considered in the discussion on the development of the standards.

## Conclusion

This paper outlines a potential approach to and consideration of factors in establishing international quality standards of care in medical travel. The OECD framework together with the key domains addressed in this paper provides policymakers with the resources to establish quality standards in medical travel. From the economic perspective, OECD may be best placed to take a leading role in developing these, having presented one of the initial calls for medical travel in the efforts to control health costs. However, to ensure the appropriate benefits for health and health services, organizations such as the WHO and other health policy bodies should be involved. As developing the standards is only the first step to their successful implementation, a wide range of stakeholders including international medical associations, insurance groups, and independent non-governmental organizations will be subsequently required to take collaborative action to support the development and further adherence to the standards. For medical travel to be assured as safe, cost-effective, and accessible for individuals in need, such standards must be in place and carried out in practice.

## Author Contributions

KR designed the study. OK, BK, CM, AS, and EH conducted the literature review and identified three main domains to be considered for the development of international quality standards in medical travel. OK, BK, CM, AS, and CD wrote the manuscript. KR and SH supervised the project and edited the manuscript.

## Conflict of Interest Statement

The authors declare that the research was conducted in the absence of any commercial or financial relationships that could be construed as a potential conflict of interest. The reviewers (NM and MM) and handling Editor declared their shared affiliation, and the handling Editor states that the process nevertheless met the standards of a fair and objective review.
